# Inflammatory Bowel Disease and Risk of Colorectal Cancer: An Overview From Pathophysiology to Pharmacological Prevention

**DOI:** 10.3389/fphar.2021.772101

**Published:** 2021-10-20

**Authors:** Marianna Lucafò, Debora Curci, Martina Franzin, Giuliana Decorti, Gabriele Stocco

**Affiliations:** ^1^ Institute for Maternal and Child Health-IRCCS Burlo Garofolo, Trieste, Italy; ^2^ Department of Medical, Surgical and Health Sciences, University of Trieste, Trieste, Italy; ^3^ Department of Life Sciences, University of Trieste, Trieste, Italy

**Keywords:** inflammatory bowel deases, colorectal cancer, inflammation, epigenetics, microbiota, immunomodulators

## Abstract

Increased risk of colorectal cancer (CRC) in inflammatory bowel disease (IBD) patients has been attributed to long-standing chronic inflammation, with the contribution of genetic alterations and environmental factors such as the microbiota. Moreover, accumulating data indicate that IBD-associated CRC (IBD-CRC) may initiate and develop through a pathway of tumorigenesis distinct from that of sporadic CRC. This mini-review summarizes the current knowledge of IBD-CRC, focusing on the main mechanisms underlying its pathogenesis, and on the important role of immunomodulators and biologics used to treat IBD patients in interfering with the inflammatory process involved in carcinogenesis.

## Introduction

**FIGURE 1:**
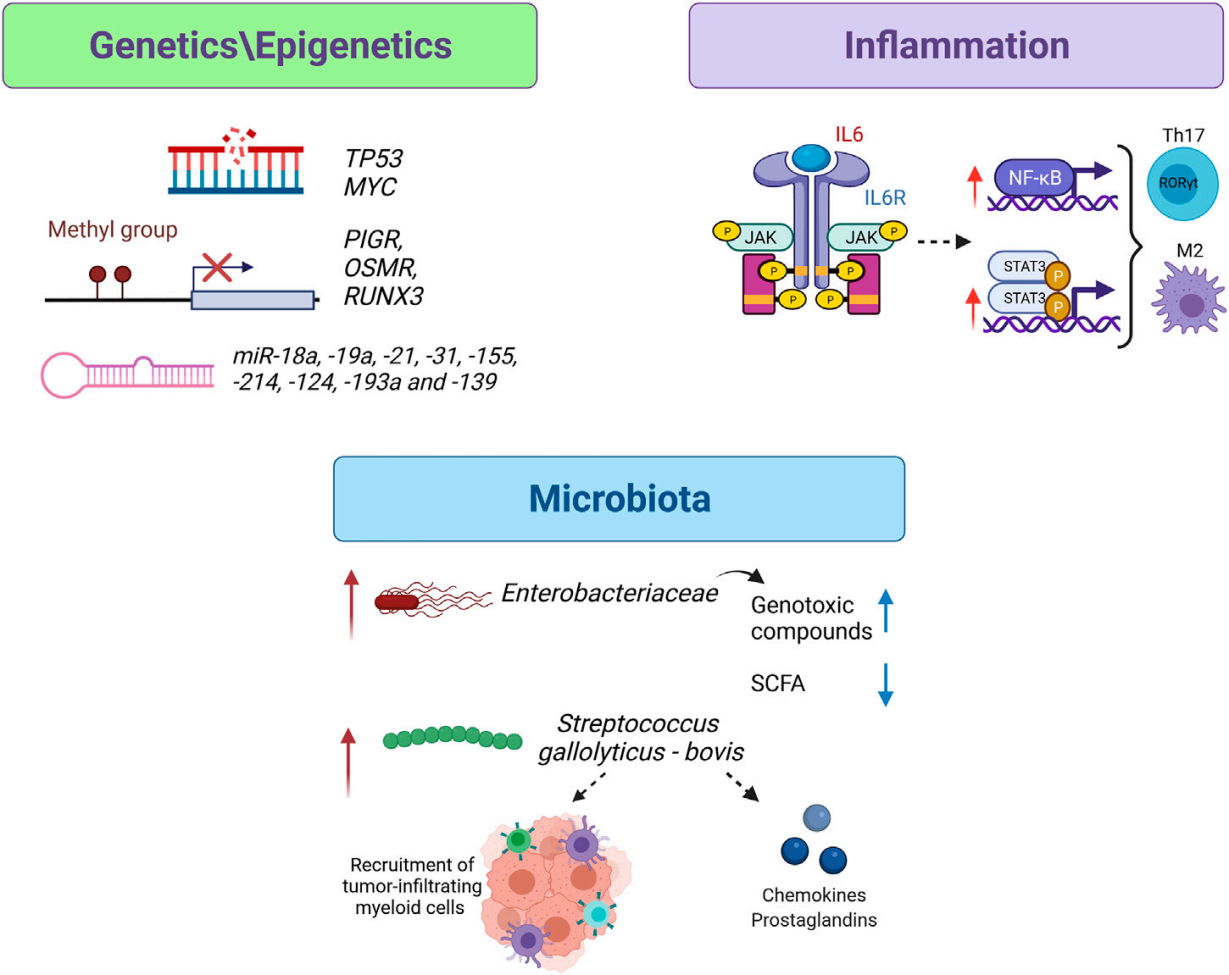
From IBD to CRC: possible key players in carcinogenesis within intestinal mucosa. Intestinal epithelial cells of IBD-CRC patients present a different frequency of somatic mutations, altered DNA methylation sites and deregulated microRNA in comparison to sCRC. The anti-apoptotic role of NF-κB in cancer cells enhanced their proliferation, promoting tumor progression. Moreover, an exacerbated activation of NF-κB `and STAT3 signaling, *via* IL6, drives M2 polarization and Th17 differentiation favouring IBD-CRC carcinogenesis. *Enterobacteriaceae* strains are responsible for the production of genotoxic compounds and for the reduction of intestinal levels of anti-inflammatory SCFA; *Streptococcus* genus could exert its tumorigenic activity through the recruitment of tumor-infiltrating myeloid cells (*S. gallolyticus*) and the release of chemokines and prostaglandins (*S. bovis*). The image was created with BioRender.com.

Inflammatory bowel disease (IBD), comprising ulcerative colitis (UC) and Crohn’s disease (CD), is a chronic relapsing inflammatory disorder associated with an increased risk of colorectal cancer (CRC) compared to the general population (Eaden et al., 2001; Bajpai et al., 2019). Interestingly, incidence rates of IBD-associated CRC (IBD-CRC) decreased over the last decades (Castaño-Milla et al., 2014; Choi et al., 2015). Several risk factors have been described, such as disease duration and extension (Ekbom et al., 1990; Eaden et al., 2001), inflammation (Rutter et al., 2004) and primary sclerosing cholangitis (Soetikno et al., 2002). Despite the limited number of studies, younger age at onset and onset of IBD in childhood seem to be associated with an increased incidence of CRC (Jess et al., 2012; Olén et al., 2020). Unlike sporadic CRC (sCRC), in patients with IBD-CRC, long-standing chronic inflammation initiates and drives tumorigenesis and important elucidation of the multiple factors involved in the process of carcinogenesis are emerging (Ullman and Itzkowitz, 2011; Baker et al., 2018). The present mini-review summarizes the recent advances in the pathophysiology of IBD-CRC, including the role of the immunomodulators currently used in the treatment of IBD (Figure 1).

## Pathogenesis of CRC in IBD Patients: Genetic and Epigenetic Bases

CRC tumorigenesis needs a tumor-initiating event that modifies normal intestinal epithelial cells by spontaneous mutation, environmental mutagens or inflammation-induced genetic and epigenetic alterations (Schmitt and Greten, 2021). Several lines of evidence suggest that IBD-CRC can result from a different mechanism of tumorigenesis in comparison to sCRC (Salk et al., 2009; Risques et al., 2011; Baker et al., 2019). The epithelial tumor tissue of IBD-CRC patients presents a lower frequency of somatic adenomatous polyposis coli (*APC)* and Kirsten rat sarcoma virus (*KRAS)* mutations, while tumor protein P53 (*TP53)* mutations and Myc proto-oncogene protein (*MYC*) amplifications are detected earlier during tumor progression in comparison to sCRC (Yaeger et al., 2016; Du et al., 2017; Chatila et al., 2020). Insights on germline alterations of IBD patients with CRC were provided for 25 patients with IBD-CRC, sequencing 39 genes potentially involved in predisposition to cancer (Biscaglia et al., 2021). Six patients (24%) presented pathogenic variants (International Agency for Research on Cancer, IARC class 4 or 5). Of these, four concerned the *APC* region, three the *MLH1* gene, and the remaining ones the *MSH2*, *MSH3*, *MUTYH*, *EPCAM*, *BRCA1*, *CHEK2*, *POLD1*, *POLE*, *CDKN2A* and *PDGFRA* genes. The onset of CRC was significantly earlier in patients carrying these variants than in patients with benign or unidentified variants.

DNA and RNA-sequencing and methylation analysis were performed in 2500 CRC cases, including 31 IBD-CRC (Rajamäki et al., 2021). As expected, somatic mutations in *APC* and *KRAS* were less frequent in IBD-CRC; a significant enrichment of somatic mutations at noncoding 5′UTR of *TP53* in IBD-CRC, resulting in low TP53 expression, was found. Aberrant promoter methylation patterns were detected exclusively in IBD-CRC in two genes related to mucosal immunity, in particular hypermethylation of polymeric immunoglobulin receptor (*PIGR)* and hypomethylation and strong overexpression of oncostatin M receptor (*OSMR*). Interestingly, increased levels of the interleukin-6 (IL-6) family member OSM and its receptor have been already detected in patients with active IBD and their presence was associated with failure of anti-tumor necrosis factor *a* (TNF) therapy (West et al., 2017), suggesting a potential role of the OSMR signaling in the molecular mechanism of IBD-associated tumorigenesis.

Chronic inflammation promotes aberrant DNA methylation in IBD, which in turn may predispose to the development of cancer (Barnicle et al., 2017; Somineni et al., 2019). A progressive increase in the percentage of methylated genes in the WNT signaling pathway from normal colon samples (n = 24) to IBD (n = 25) to IBD-CRC (n = 16) was observed, indicating their potential involvement during cancer development (Dhir et al., 2008). In particular, methylation of *APC1A*, *APC2*, *SFRP1*, and *SFRP2* genes characterized the progression from IBD to IBD-CRC, indicating their potential role as biomarkers for early detection of cancer in IBD patients. These results have recently been validated in an additional cohort of UC CRC (Beggs et al., 2019).

The methylation status of 10 candidate genes involved in tumor suppression, cell-cycle regulation, and aging, in UC–CRC tumors and non-neoplastic tissues from both UC-CRC and UC patients (n = 114 for each) was analyzed (Garrity-Park et al., 2010). Methylation of *RUNX3*, *MINT1*, and *COX-2* genes in non-neoplastic tissue was significantly associated with UC-CRC, suggesting their role as potential indicators of carcinogenesis (Garrity-Park et al., 2010). An altered methylation status of *RUNX3* in the non-neoplastic sections of UC-CRC was also observed by Scarpa and others (Scarpa et al., 2016).

Among the epigenetic factors, emerging data have implicated the altered expression of specific microRNAs in IBD-associated tumorigenesis: miR-18a, -19a, -21, -31, -155 and -214 were upregulated in IBD-CRC colon tissues compared to healthy controls while miR-124, -193a and -139 were downregulated (Bocchetti et al., 2021), however, further prospective studies on large cohort of patients are needed.

## Inflammation and Tumorigenesis in IBD-CRC

The innate and adaptive immune system cells play an important role in the onset of IBD-CRC. Crosstalk between these cell types occurs mainly through a network of cytokines that drive and maintain inflammation and contribute to tumorigenesis *via* oxidative stress, epithelial cell proliferation, and angiogenesis (Long et al., 2017). In Table 1 is reported a brief summary of the effects of different inflammation-related molecules in IBD-CRC.

**TABLE 1:** cytokines and/or pro-inflammatory molecules in IBD-CRC.

Molecule	Function in IBD-CRC	Reference
TNF-α	Activates oncogenic signaling pathways in epithelial cells, such as Wnt and NF-κB, that maintain a pro-inflammatory environment favoring tumor progression and angiogenesis.	Grivennikov and Karin, (2011)
IL-1β	Induces tumor cell proliferation and leads to Wnt signaling pathway activation.	Hnatyszyn et al. (2019)
IL-6	Critical for long-standing inflammation, for the recruitment and activation of Th17 cells and the inhibition of the regulatory T cells functions. The ability of IL-6 to activate STAT3 in epithelial cells is critical for its pro-tumorigenic activity.	Grivennikov et al. (2009)
IL-8	This chemokine was associated with increased metastatic and angiogenic potential in a mouse model of IBD-CRC.	Luo and Zhang, (2017)
IL-21	In mouse intestinal epithelial cells, IL-21 increases the risk of IBD-CRC by enhancing the expression of induced cytidine deaminase gene which deaminates cytosine residues to cause cytosine-to-thymine transitions.	Araki et al. (2019)
IL-22	Induces proliferation of enterocytes and dysplasia in a mouse model of IBD-CRC. IL-22 induces the nitric oxide synthase that leads to nitric oxide production within crypt epithelial cells driving DNA damage and carcinogenesis.	Wang et al. (2017)
COX-2	Overexpression of COX-2 contributes to increased proliferation, angiogenesis and resistance to apoptosis favoring tumour initiation and progression. The release of proinflammatory cytokines induced and maintained COX-2 expression and leads to the transition from acute to chronic inflammation. The use of COX-2 inhibitors reduces IBD-CRC development in mice thanks to the inhibition of cell proliferation, the reduction of β-catenin activation, COX-2 activity and nitric oxide production.	Kohno et al. (2005); Hnatyszyn et al. (2019)
Prostaglandins	Prostaglandin E2/EP favors IBD-CRC development by switching the phenotype of macrophages and neutrophils to pro-tumor, increasing cellular migration through the up-regulation of vascular endothelial growth factor receptor-1 signaling and by activating NF-κB.	Mizuno et al. (2019)

Among these molecules, the macrophage migration inhibitory factor (MIF), a pleiotropic cytokine that drives cellular proliferation and regulates the migration and activation state of immune cells, seems to be relevant. The pathophysiological role of MIF in a wide range of inflammatory diseases, among which IBD, was already demonstrated (Nishihira and Mitsuyama, 2009). Increased MIF in macrophages in a CRC mouse model was demonstrated, and loss of MIF expression protects mice during tumor initiation (Pacheco-Fernández et al., 2019; Klemke et al., 2021). In cancer cells from CRC patients and in an acute colitis-CRC mouse model, a tumor-specific elevation of MIF expression was demonstrated (Klemke et al., 2021). The heat shock protein 90 (HSP90) chaperone machinery stabilizes and protects MIF from degradation and supports tumor progression *via* macrophage recruitment and angiogenesis.

In the context of inflammation and CRC, the most investigated pathways are the nuclear factor *κ* light-chain enhancer (NF-κB) and IL-6/signal transducer and activator of transcription 3 (STAT3) and STAT6 signaling pathways (Grivennikov and Karin, 2011).

NF-κB plays an important role in tumorigenic process by several mechanisms: promotes the production of reactive oxygen and nitrogen species which induce DNA damage, causes chromosomal instability and epigenetic changes (Grivennikov and Karin, 2011). Furthermore, by stimulating the production of inflammatory cytokines and growth factors, NF-κB enhances the proliferation of tumor progenitor cells, favoring tumor progression. This effect is also enhanced by the anti-apoptotic role of NF-κB; in fact in a IBD-CRC mouse model, it was demonstrated that NF-κB suppresses apoptosis through the induction of the anti-apoptotic protein B-cell lymphoma-extra large (Bcl-xL) (Greten et al., 2004). The NF-κB pathway results aberrantly activated in most of colitis-associated tumors and is involved in the expression of pro-inflammatory genes including *COX-2*, *TNF-* *α* and *IL-6* (Gambhir et al., 2015).

The impaired regulation of NF-κB in tumor cells is also mediated by STAT3 which prompts the retention of NF-κB into the nucleus and hence amplifies its effect during the tumorigenic process and increases the interactions and communication between cancer cells and the microenvironment (Onizawa et al., 2009). The pro-oncogenic effects of STAT3 are mostly evident following inactivation of the negative regulators of IL-6 signaling, such as the suppressor of cytokine signaling 3 (SOCS3), that leads to an increased phosphorylation of protein kinase B (AKT), and NF-κB, initiating the disease process in patients that will progress towards IBD-CRC (Johnson et al., 2018). An increased IL-6/p-STAT3 signaling in dysplasia and colon cancer was demonstrated (Li et al., 2010). SOCS3 expression is reduced during progression from active UC to IBD-CRC and the altered methylation of SOCS3 may be involved in tumor progression increasing STAT3 signaling.

Another mechanism by which STAT3 promotes tumor progression is by favoring immune cell recruitment *via* the sphingosine-1-phosphate (S1P) signaling (Liang et al., 2013). S1P is formed by two related sphingosine kinases, SphK1 and SphK2, and it has already been demonstrated that SphK1 and intracellular S1P maintain a persistent activation of NF-κB and STAT3 pathways that lead to IBD-CRC development (Kawamori et al., 2009; Liang et al., 2013). Interestingly, in mice, the knockout of SphK2 increased SphK1 and S1PR1 expression, providing a pro-inflammatory environment through the secretion of IL-6 and favoring the infiltration of macrophages and T cells into tumor tissues.

Being the most abundant immune cells in tumor microenvironment, tumor-associated macrophages might be critical players in IBD-CRC progression. The role of Wnt5a, a member of the Wnt family, was already assessed in CRC: Wnt5a stimulates macrophages to produce IL-10 through the activation of STAT3 signaling pathways, crucial events for the M2 tumorigenic phenotype (Liu et al., 2020). Wnt signaling has several functions in proliferation, differentiation, migration, and survival and is regulated also by the NF-κB pathway (Du and Geller, 2010). A crosstalk between Wnt/β-catenin and NF-κB signaling pathways can significantly influence the progression of inflammation and the onset of IBD-CRC.

A recent work demonstrated for the first time, using a mouse model of IBD-CRC, that the M2 macrophage polarization could be altered by genetic inactivation of the MAPK-activated protein kinase 2 (MK2), resulting in delayed tumor progression (Suarez-Lopez et al., 2020).

Other cell types involved in cancer-associated inflammation include natural killers, T-helpers, monocytes and regulatory T-cells. During IBD and progression to dysplasia, the regulatory T cells, expressing the Th17-related transcription factor RORγt, increase in the tumor and peripheral blood of individuals with IBD-CRC, releasing pro-inflammatory cytokines (Quandt et al., 2021). Authors linked this phenotype to enhanced Wnt–β-catenin signaling, inducing pro-inflammatory cytokine production and RORγt expression in Treg cells. In particular using a mouse model of IBD-CRC, they demonstrated that the binding of the β-catenin interacting partner, TCF-1, to DNA overlapped with Foxp3 binding at active enhancer regions of pro-inflammatory genes. As a consequence of Wnt–β-catenin activation, new accessible chromatin sites in these pro-inflammatory genes were generated, leading to their upregulated expression. Enhanced β-catenin binding to TCF-1 may alter the TCF-1–Foxp3-dependent regulation of these genes. In particular, pathway enrichment analysis revealed that co-binding of TCF-1 and Foxp3 increases the accessibility and transcription of genes involved in Th17 differentiation and T cell activation pathways such as *IL-17*, *IFN-γ* and *TNF*.

## Role of Microbiota in the Pathogenesis of IBD-CRC

Although the exact mechanism of inflammation-associated carcinogenesis is still not completely known, the contribution of the gut microbiota, especially of some pathogenic bacterial species, seems to be relevant. There is a general consensus on the relationship between the gut microbiota and the immune system: microbes, through pathogen-associated molecular patterns (PAMPs), are capable to communicate with pattern recognition receptors (PRRs) in the innate immune system, such as Toll-like receptors (TLRs), retinoic acid-inducible gene I-like receptors (RLRs) and nucleotide-binding oligomerization domain-receptors (NLRs), and to trigger the immune response (Mogensen, 2009; Lu et al., 2018). Interestingly, the expression of TLR4 is strongly upregulated in colonic tissues of BALB/c mice treated with azoxymethane/dextran sodium sulphate (AOM/DSS) to induce IBD-CRC, and blocking TLR4 signaling slows the development of the tumor (Pastille et al., 2021). In addition, downregulation of the *TLR2* gene inhibits the proliferation of IBD-CRC. In particular, knocking out the *TLR2* gene in mice treated with 1,2-dimethylhydrazine-dextran sodium sulphate, reduced the shortening of colorectal length, the number and volume of tumors, the pathological score and tumor severity. Furthermore, knocking down the *TLR2* gene in the colorectal cancer cell lines HCT116 and HT29 inhibited their proliferation (Meng et al., 2020). It is noteworthy that also the deficient stimulation of other PRRs, such as NOD2, leads to a higher risk of IBD-CRC: in particular, NOD2^−/−^ mice, treated with AOM to induce IBD-CRC, presented an increase in the number and size of tumors (Couturier-Maillard et al., 2013).

Moreover, upon stimulation with PAMPs, the NF-κB pathway and Wnt signaling, mentioned before for their role in the inflammation and proliferation processes leading to tumorigenesis, can be activated (Santaolalla et al., 2013; Peng et al., 2020).

Growing evidence confirms the association between IBD and the alteration of the composition of gut microbiota, sometimes referred as dysbiosis (Kang and Martin, 2017). Overgrowth of specific bacterial species at the expense of commensals is related not only to IBD but also to the development of CRC (Kang and Martin, 2017; Fan et al., 2021).

Nevertheless, to the author’s knowledge, only one study investigated the gut microbiota composition in IBD-CRC patients (Richard et al., 2018), examining the gut microbiota from colonic mucosa of 10 healthy subjects (HS), 10 patients suffering from sCRC and seven patients affected by IBD-CRC. The bacterial microbiota of IBD-CRC patients had a reduced diversity compared to HS and a composition different from that of sCRC patients. In particular, when compared to HS, IBD-CRC patients have a decreased abundance in Firmicutes and Bacteroidetes and an increase in Proteobacteria; instead, *Bradyrhizobiaceae* and *Enterobacteriaceae* families, among Proteobacteria phylum, were overexpressed in the mucosa of IBD-CRC in comparison to sCRC patients (Richard et al., 2018). Interestingly, the *Bradyrhizobiaceae* and *Enterobacteriaceae* families, also abundantly proliferate in the mucosal and luminal gut of IBD patients, suggesting that the predominance of these microorganisms could be due to the pre-existing disease and that they could have a pathogenetic role in the inflammation-associated carcinogenesis (Swidsinski et al., 2002; Kaakoush et al., 2012; Wang et al., 2015).

The proliferation of *Enterobacteriaceae* is also associated with a lower concentration in short chain fatty acids (SCFAs); indeed, these metabolites counteract the competitive edge that O_2_ and NO_2_ give to this bacterial family during growth (Sorbara et al., 2019). Indeed, SCFAs, such as acetate, propionate and butyrate, are produced through the anaerobic fermentation of non-digestible dietary fibers by specific bacterial species, such as *Faecalibacterium prausnitzii*, *Clostridium leptum*, *Eubacterium rectale* and some *Roseburia* species, belonging to Firmicutes, whose abundance and diversity decreases in the gut microbiota of both IBD and IBD-CRC patients (Tan et al., 2014; Richard et al., 2018; Parada Venegas et al., 2019). SCFAs are a source of energy for colonocytes, elicit anti-inflammatory effects and exert antitumorigenic activity (Tan et al., 2014; Parada Venegas et al., 2019). In particular, SCFAs exert their anti-inflammatory properties binding to their FFAR2 and HCAR2 receptors, expressed on intestinal epithelial and immune cells, and thus inducing neutrophil chemotaxis to inflammatory sites, stimulating intestinal IgA secretion towards pathogenic bacteria and increasing the secretion of IL-18, which promotes gut epithelial integrity, repair and intestinal homeostasis, *via* inflammasome activation and IL-10, which promotes the differentiation of Treg cells (Tian et al., 2018). Instead, the antitumorigenic activity of SCFAs, especially butyrate, has been mainly attributed to the inhibition of the proliferation and the induction of apoptosis in cancer cells achieved through the alteration of gene transcription by inhibiting the activity of histone deacetylase (Tian et al., 2018). The administration of a mixture of SCFAs attenuated colonic inflammation and improved disease activity index, suppressing the expression of the proinflammatory cytokines IL-6, TNFα and IL-17 in BALB/c mice with AOM/DSS-induced CRC; the mixture also reduced the tumor incidence and size (Tian et al., 2018).

Among the *Enterobacteriaceae* family, *Escherichia coli* utilizes virulence factors, such as colibactin, a genotoxic compound, that promotes tumor growth in a xenograft mouse model and in mice with functioning autophagy, who lack for this reason genetic susceptibility for carcinogenesis, after treatment with AOM/DSS to induce CRC (Dalmasso et al., 2014; Salesse et al., 2021). Indeed, colibactin alkylates DNA and induces double-stranded breaks, playing thus a pro-tumorigenic role (Yang et al., 2020). Interestingly, a higher prevalence of colibactin-producing *E. coli* in patients affected by IBD compared to healthy individuals was demonstrated: inflammation could cause the upregulation of the *colibactin* gene and also facilitates the colonization of the mucosa by *E. coli,* leading to an increase in colibactin-induced DNA damage and allowing this bacterial strain to exert its carcinogenic activity (Yang et al., 2020).

Furthermore, a difference in microbial composition between the tumor and tumor-surrounding area, even if less pronounced than in sCRC, was evidenced; indeed, the *Streptococcus* genus was found to be more abundant in the IBD-CRC microbiota compared to the healthy adjacent mucosa (Richard et al., 2018). *Streptococcus* species, representative of the bacterial population of the mucosa and of the lumen of IBD patients, are associated with tumorigenesis (Biarc et al., 2004; Santoru et al., 2017; Zhang et al., 2018; Lo Presti et al., 2019). For instance, *S. gallolyticus* allows the tumor progression in C57BL/6 mice with AOM/DSS-induced CRC through the recruitment of tumor-infiltrating myeloid cells which can inhibit competence of T cells and increase proinflammatory cytokines (Zhang et al., 2018). Moreover, 12 proteins isolated from *S. bovis* are able to trigger the release of chemokines and prostaglandins in both human epithelial colonic Caco-2 cells and in rat colonic mucosa, and to promote pre-neoplastic lesions in AOM-treated rats (Biarc et al., 2004).

Investigating the gut microbiota in the AOM-DSS mouse model of IBD-CRC, similarly to what was encountered for IBD-CRC patients the microbial community was drastically altered by chronic colitis: in particular, in addition to *Lactobacillus hamster*, *Bacteroides uniformis* and *Bacteroides ovatus*, also *Streptococcus luteciae*, belonging to *Streptococcus* genus mentioned above for its pathogenic role, increases (Liang et al., 2014).

## Chemopreventive Effects of Therapies for IBD

Since several observations support the important role of inflammation in the development of IBD-CRC, the use of anti-inflammatory and immunosuppressant drugs in IBD can reasonably reduce inflammation in the gut and consequently the risk of inflammation-related cancers.

The chemopreventive effect of 5-aminosalicylic acid (5-ASA) in IBD patients has been widely studied even though the results remain conflicting (Terdiman et al., 2007; Bernstein et al., 2011; Carrat et al., 2017). A systematic literature search including 164 studies and meta-analyses to identify all prognostic factors for advanced CRC in patients with IBD (Wijnands et al., 2021), showed that patients who received 5-ASA had a lower risk of advanced CRC. In a systematic review, a protective role of 5-ASA against CRC in UC patients in clinical-based studies but not in population-based studies was shown (Qiu et al., 2017). In IBD patients, 5-ASA at a dosage ≥1.2 g/day showed higher protective effects against CRC than at dosages <1.2 g/day. Interestingly, a recent observational study provided molecular evidence of changes in genes related to the carcinogenesis pathways such as *CDC25A, CXCL10, IL8, NF-κB*, and *Ki-67* in colonic biopsies of 62 UC patients during long-term 5-ASA maintenance therapy; these changes may contribute to the chemopreventive effects observed in UC patients (Bajpai et al., 2019).

One of the most recent systematic review and meta-analysis, including 11 cohort and 16 case-control studies and involving 95,397 patients, highlighted that the use of thiopurines, azathioprine and mercaptopurine, was associated with a reduced risk of CRC; this chemopreventive effect was confirmed in patients with long disease duration (Beaugerie et al., 2013) but not in those with extensive colitis or primary sclerosing cholangitis (Zhu et al., 2018). Studies conducted on CESAME (19,486 patients) and ENEIDA (831 patients) cohorts confirmed that the risk for CRC is lower among IBD patients receiving thiopurine therapy (Beaugerie et al., 2013; Gordillo et al., 2015). In a well-established murine model, the thiopurine thioguanine inhibits colitis-associated cancer by decreasing β-catenin activation/nuclear translocation, providing important evidence in support of the potential therapeutic utility of this class of drugs (Sheng et al., 2021).

The impact of biological drugs on IBD-CRC development has yet to be definitely confirmed and long-term follow-up studies will be extremely important. Considering the role of TNFα in the initiation and progression of IBD-CRC (Popivanova et al., 2008; Wilson, 2008) the use of anti-TNFα agents may be useful in preventing CRC in patients with IBD (Biancone et al., 2009). A large-scale database study showing the inverse association of CRC with anti-TNFα therapy in the IBD population (225,090 CD and 188,420 UC) was recently published (Alkhayyat et al., 2021). In this study, patients with IBD who received any of the anti-TNFα agents and those who received combined treatment (anti-TNFs plus immunomodulators) had a lower risk of developing CRC. The relationship between anti-TNFα and CRC in IBD is also supported by the results obtained in animal models treated with infliximab in which CRC carcinogenesis associated with chronic colitis was reduced (Kim et al., 2010).

## Conclusion

In summary, this mini-review summarizes the recent advances in the knowledge of the pathophysiology of IBD-CRC, a complex disease associated with multifactorial causes. Inflammatory pathways seem to be the major drivers of tumorigenesis in IBD patients even if the mechanisms that link inflammation and carcinogenesis remain not well characterized in patients. In this context, development of therapies targeting specific proinflammatory cytokines involved in tumorigenesis can provide a novel approach to prevent tumor initiation or progression. Further studies with large numbers of subjects are needed to address the existing gaps in the knowledge of the role of epigenetics in the process of carcinogenesis and to validate the predictive power and clinical value of the data collected so far. The identification of predictive and prognostic epigenetic markers could favor an early detection of IBD patients with increased risk of CRC. These analyses could also consider purified cellular populations, in particular epithelial cells.

Since the mucosal associated microbiota of IBD-CRC patients is characterized by the overgrowth of bacterial species playing a role in the pathogenesis of IBD-CRC, it could be assumed that increasing the levels of beneficial bacteria with probiotics could enhance the levels of the anti-inflammatory bacterial products SCFAs, restore the equilibrium and possibly ameliorate IBD-CRC condition. In addition, IBD-CRC may be at least partially prevented through mucosal healing of intestinal lesions, and the power of the potential anti-tumor effects of IBD drugs should be evaluated by rigorous prospective studies in the near future.

## References

[B1] Alkhayyat M. Abureesh M. Gill A. Khoudari G. Abou Saleh M. Mansoor E. (2021). Lower Rates of Colorectal Cancer in Patients with Inflammatory Bowel Disease Using Anti-TNF Therapy. Inflamm. Bowel Dis. 27 (7), 1052–1060. 10.1093/ibd/izaa252 33051651

[B2] Araki A. Jin L. Nara H. Takeda Y. Nemoto N. Gazi M. Y. (2019). IL-21 Enhances the Development of Colitis-Associated Colon Cancer: Possible Involvement of Activation-Induced Cytidine Deaminase Expression. J. Immunol. 202 (11), 3326–3333. 10.4049/jimmunol.1800550 31019062

[B3] Bajpai M. Seril D. N. Van Gurp J. Geng X. Alvarez J. Minacapelli C. D. (2019). Effect of Long-Term Mesalamine Therapy on Cancer-Associated Gene Expression in Colonic Mucosa of Patients with Ulcerative Colitis. Dig. Dis. Sci. 64 (3), 740–750. 10.1007/s10620-018-5378-8 30478770

[B4] Baker A. M. Cross W. Curtius K. Al Bakir I. Choi C. R. Davis H. L. (2019). Evolutionary History of Human Colitis-Associated Colorectal Cancer. Gut 68 (6), 985–995. 10.1136/gutjnl-2018-316191 29991641 PMC6580738

[B5] Baker K. T. Salk J. J. Brentnall T. A. Risques R. A. (2018). Precancer in Ulcerative Colitis: the Role of the Field Effect and its Clinical Implications. Carcinogenesis 39 (1), 11–20. 10.1093/carcin/bgx117 29087436 PMC6248676

[B6] Barnicle A. Seoighe C. Greally J. M. Golden A. Egan L. J. (2017). Inflammation-associated DNA Methylation Patterns in Epithelium of Ulcerative Colitis. Epigenetics 12 (8), 591–606. 10.1080/15592294.2017.1334023 28557546 PMC5687324

[B7] Beaugerie L. Svrcek M. Seksik P. Bouvier A. M. Simon T. Allez M. (2013). Risk of Colorectal High-Grade Dysplasia and Cancer in a Prospective Observational Cohort of Patients with Inflammatory Bowel Disease. Gastroenterology 145 (1), 166. 10.1053/j.gastro.2013.03.044 23541909

[B8] Beggs A. D. Mehta S. Deeks J. J. James J. D. Caldwell G. M. Dilworth M. P. (2019). Validation of Epigenetic Markers to Identify Colitis Associated Cancer: Results of Module 1 of the ENDCAP-C Study. EBioMedicine 39, 265–271. 10.1016/j.ebiom.2018.11.034 30473377 PMC6355942

[B9] Bernstein C. N. Nugent Z. Blanchard J. F. (2011). 5-aminosalicylate Is Not Chemoprophylactic for Colorectal Cancer in IBD: a Population Based Study. Am. J. Gastroenterol. 106 (4), 731–736. 10.1038/ajg.2011.50 21407180

[B10] Biancone L. Petruzziello C. Calabrese E. Zorzi F. Naccarato P. Onali S. (2009). Long-term Safety of Infliximab for the Treatment of Inflammatory Bowel Disease: Does Blocking TNFalpha Reduce Colitis-Associated Colorectal Carcinogenesis? Gut 58 (12), 1703. 10.1136/gut.2008.176461 19923350

[B11] Biarc J. Nguyen I. S. Pini A. Gossé F. Richert S. Thiersé D. (2004). Carcinogenic Properties of Proteins with Pro-inflammatory Activity from Streptococcus Infantarius (Formerly S.Bovis). Carcinogenesis 25 (8), 1477–1484. 10.1093/carcin/bgh091 14742316

[B12] Biscaglia G. Latiano A. Castellana S. Fontana R. Gentile A. Latiano T. (2021). Germline Alterations in Patients with IBD-Associated Colorectal Cancer. Inflamm. Bowel Dis., izab195. 10.1093/ibd/izab195 34347074

[B13] Bocchetti M. Ferraro M. G. Ricciardiello F. Ottaiano A. Luce A. Cossu A. M. (2021). The Role of microRNAs in Development of Colitis-Associated Colorectal Cancer. Int. J. Mol. Sci. 22 (8), 3967. 10.3390/ijms22083967 33921348 PMC8068787

[B14] Carrat F. Seksik P. Colombel J. F. Peyrin-Biroulet L. Beaugerie L. (2017). The Effects of Aminosalicylates or Thiopurines on the Risk of Colorectal Cancer in Inflammatory Bowel Disease. Aliment. Pharmacol. Ther. 45 (4), 533–541. 10.1111/apt.13897 27995656

[B15] Castaño-Milla C. Chaparro M. Gisbert J. P. (2014). Systematic Review with Meta-Analysis: the Declining Risk of Colorectal Cancer in Ulcerative Colitis. Aliment. Pharmacol. Ther. 39 (7), 645–659. 10.1111/apt.12651 24612141

[B16] Chatila W. K. Walch H. S. Benhamida J. Hechtman J. F. Barriga F. M. Kundra R. (2020). Genomic Alterations in Colitis-Associated Cancers in Comparison to Those Found in Sporadic Colorectal Cancer and Present in Precancerous Dysplasia. Jco 38 (4_Suppl. l), 191. 10.1200/jco.2020.38.4_suppl.191

[B17] Choi C. H. Rutter M. D. Askari A. Lee G. H. Warusavitarne J. Moorghen M. (2015). Forty-Year Analysis of Colonoscopic Surveillance Program for Neoplasia in Ulcerative Colitis: An Updated Overview. Am. J. Gastroenterol. 110 (7), 1022–1034. 10.1038/ajg.2015.65 25823771 PMC4517513

[B18] Couturier-Maillard A. Secher T. Rehman A. Normand S. De Arcangelis A. Haesler R. (2013). NOD2-mediated Dysbiosis Predisposes Mice to Transmissible Colitis and Colorectal Cancer. J. Clin. Invest. 123 (2), 700–711. 10.1172/JCI62236 23281400 PMC3561825

[B19] Dalmasso G. Cougnoux A. Delmas J. Darfeuille-Michaud A. Bonnet R. (2014). The Bacterial Genotoxin Colibactin Promotes colon Tumor Growth by Modifying the Tumor Microenvironment. Gut microbes 5 (5), 675–680. 10.4161/19490976.2014.969989 25483338 PMC4615906

[B20] Dhir M. Montgomery E. A. Glöckner S. C. Schuebel K. E. Hooker C. M. Herman J. G. (2008). Epigenetic Regulation of WNT Signaling Pathway Genes in Inflammatory Bowel Disease (IBD) Associated Neoplasia. J. Gastrointest. Surg. 12 (10), 1745–1753. 10.1007/s11605-008-0633-5 18716850 PMC3976145

[B21] Du L. Kim J. J. Shen J. Chen B. Dai N. (2017). KRAS and TP53 Mutations in Inflammatory Bowel Disease-Associated Colorectal Cancer: a Meta-Analysis. Oncotarget 8 (13), 22175–22186. 10.18632/oncotarget.14549 28077799 PMC5400656

[B22] Du Q. Geller D. (2010). Cross-Regulation between WNT and NF-κB Signaling Pathways. Immunopathol Dis. Therap. 1, 155–181. 10.1615/ForumImmunDisTher.v1.i3 PMC3114374 21686046

[B23] Eaden J. A. Abrams K. R. Mayberry J. F. (2001). The Risk of Colorectal Cancer in Ulcerative Colitis: a Meta-Analysis. Gut 48 (4), 526–535. 10.1136/gut.48.4.526 11247898 PMC1728259

[B24] Ekbom A. Helmick C. Zack M. Adami H. O. (1990). Ulcerative Colitis and Colorectal Cancer. A Population-Based Study. N. Engl. J. Med. 323 (18), 1228–1233. 10.1056/NEJM199011013231802 2215606

[B25] Fan X. Jin Y. Chen G. Ma X. Zhang L. (2021). Gut Microbiota Dysbiosis Drives the Development of Colorectal Cancer. Digestion 102 (4), 508–515. 10.1159/000508328 32932258

[B26] Gambhir S. Vyas D. Hollis M. Aekka A. Vyas A. (2015). Nuclear Factor Kappa B Role in Inflammation Associated Gastrointestinal Malignancies. World J. Gastroenterol. 21 (11), 3174–3183. 10.3748/wjg.v21.i11.3174 25805923 PMC4363746

[B27] Garrity-Park M. M. Loftus E. V. Jr. Sandborn W. J. Bryant S. C. Smyrk T. C. (2010). Methylation Status of Genes in Non-neoplastic Mucosa from Patients with Ulcerative Colitis-Associated Colorectal Cancer. Am. J. Gastroenterol. 105 (7), 1610–1619. 10.1038/ajg.2010.22 20160714

[B28] Gordillo J. Cabré E. Garcia-Planella E. Ricart E. Ber-Nieto Y. Márquez L. (2015). Thiopurine Therapy Reduces the Incidence of Colorectal Neoplasia in Patients with Ulcerative Colitis. Data from the ENEIDA Registry. J. Crohns Colitis 9 (12), 1063–1070. 10.1093/ecco-jcc/jjv145 26351379

[B29] Greten F. R. Eckmann L. Greten T. F. Park J. M. Li Z. W. Egan L. J. (2004). IKKbeta Links Inflammation and Tumorigenesis in a Mouse Model of Colitis-Associated Cancer. Cell 118 (3), 285–296. 10.1016/j.cell.2004.07.013 15294155

[B30] Grivennikov S. Karin E. Terzic J. Mucida D. Yu G. Y. Vallabhapurapu S. (2009). IL-6 and Stat3 Are Required for Survival of Intestinal Epithelial Cells and Development of Colitis-Associated Cancer. Cancer Cell 15 (2), 103–113. 10.1016/j.ccr.2009.01.001 19185845 PMC2667107

[B31] Grivennikov S. I. Karin M. (2011). Inflammatory Cytokines in Cancer: Tumour Necrosis Factor and Interleukin 6 Take the Stage. Ann. Rheum. Dis. 70 (Suppl. 1), i104–8. 10.1136/ard.2010.140145 21339211

[B32] Hnatyszyn A. Hryhorowicz S. Kaczmarek-Ryś M. Lis E. Słomski R. Scott R. J. (2019). Colorectal Carcinoma in the Course of Inflammatory Bowel Diseases. Hered. Cancer Clin. Pract. 17, 18. 10.1186/s13053-019-0118-4 31338130 PMC6626407

[B33] Jess T. Rungoe C. Peyrin-Biroulet L. (2012). Risk of Colorectal Cancer in Patients with Ulcerative Colitis: a Meta-Analysis of Population-Based Cohort Studies. Clin. Gastroenterol. Hepatol. 10 (6), 639–645. 10.1016/j.cgh.2012.01.010 22289873

[B34] Johnson D. E. O'Keefe R. A. Grandis J. R. (2018). Targeting the IL-6/JAK/STAT3 Signalling axis in Cancer. Nat. Rev. Clin. Oncol. 15 (4), 234–248. 10.1038/nrclinonc.2018.8 29405201 PMC5858971

[B35] Kaakoush N. O. Day A. S. Huinao K. D. Leach S. T. Lemberg D. A. Dowd S. E. (2012). Microbial Dysbiosis in Pediatric Patients with Crohn's Disease. J. Clin. Microbiol. 50 (10), 3258–3266. 10.1128/JCM.01396-12 22837318 PMC3457451

[B36] Kang M. Martin A. (2017). Microbiome and Colorectal Cancer: Unraveling Host-Microbiota Interactions in Colitis-Associated Colorectal Cancer Development. Semin. Immunol. 32, 3–13. 10.1016/j.smim.2017.04.003 28465070

[B37] Kawamori T. Kaneshiro T. Okumura M. Maalouf S. Uflacker A. Bielawski J. (2009). Role for Sphingosine Kinase 1 in colon Carcinogenesis. FASEB J. 23 (2), 405–414. 10.1096/fj.08-117572 18824518 PMC2630788

[B38] Kim Y. J. Hong K. S. Chung J. W. Kim J. H. Hahm K. B. (2010). Prevention of Colitis-Associated Carcinogenesis with Infliximab. Cancer Prev. Res. 3 (10), 1314–1333. 10.1158/1940-6207.CAPR-09-0272 20736334

[B39] Klemke L. De Oliveira T. Witt D. Winkler N. Bohnenberger H. Bucala R. (2021). Hsp90-stabilized MIF Supports Tumor Progression via Macrophage Recruitment and Angiogenesis in Colorectal Cancer. Cell Death Dis. 12 (2), 155. 10.1038/s41419-021-03426-z 33542244 PMC7862487

[B40] Kohno H. Suzuki R. Sugie S. Tanaka T. (2005). Suppression of Colitis-Related Mouse colon Carcinogenesis by a COX-2 Inhibitor and PPAR Ligands. BMC Cancer 5 (1), 46. 10.1186/1471-2407-5-46 15892897 PMC1156872

[B41] Li Y. de Haar C. Chen M. Deuring J. Gerrits M. M. Smits R. (2010). Disease-related Expression of the IL6/STAT3/SOCS3 Signalling Pathway in Ulcerative Colitis and Ulcerative Colitis-Related Carcinogenesis. Gut 59 (2), 227–235. 10.1136/gut.2009.184176 19926618

[B42] Liang J. Nagahashi M. Kim E. Y. Harikumar K. B. Yamada A. Huang W. C. (2013). Sphingosine-1-phosphate Links Persistent STAT3 Activation, Chronic Intestinal Inflammation, and Development of Colitis-Associated Cancer. Cancer Cell 23 (1), 107–120. 10.1016/j.ccr.2012.11.013 23273921 PMC3578577

[B43] Liang X. Li H. Tian G. Li S. (2014). Dynamic Microbe and Molecule Networks in a Mouse Model of Colitis-Associated Colorectal Cancer. Sci. Rep. 4, 4985. 10.1038/srep04985 24828543 PMC4021569

[B44] Liu Q. Yang C. Wang S. Shi D. Wei C. Song J. (2020). Wnt5a-induced M2 Polarization of Tumor-Associated Macrophages via IL-10 Promotes Colorectal Cancer Progression. Cell Commun Signal 18 (1), 51. 10.1186/s12964-020-00557-2 32228612 PMC7106599

[B45] Lo Presti A. Zorzi F. Del Chierico F. Altomare A. Cocca S. Avola A. (2019). Fecal and Mucosal Microbiota Profiling in Irritable Bowel Syndrome and Inflammatory Bowel Disease. Front. Microbiol. 10, 1655. 10.3389/fmicb.2019.01655 31379797 PMC6650632

[B46] Long A. G. Lundsmith E. T. Hamilton K. E. (2017). Inflammation and Colorectal Cancer. Curr. Colorectal Cancer Rep. 13 (4), 341–351. 10.1007/s11888-017-0373-6 29129972 PMC5678998

[B47] Lu Y. Li X. Liu S. Zhang Y. Zhang D. (2018). Toll-like Receptors and Inflammatory Bowel Disease. Front. Immunol. 9, 72. 10.3389/fimmu.2018.00072 29441063 PMC5797585

[B48] Luo C. Zhang H. (2017). The Role of Proinflammatory Pathways in the Pathogenesis of Colitis-Associated Colorectal Cancer. Mediators Inflamm. 2017, 5126048. 10.1155/2017/5126048 28852270 PMC5568615

[B49] Meng S. Li Y. Zang X. Jiang Z. Ning H. Li J. (2020). Effect of TLR2 on the Proliferation of Inflammation-Related Colorectal Cancer and Sporadic Colorectal Cancer. Cancer Cel Int. 20, 95. 10.1186/s12935-020-01184-0 PMC7104506 32256204

[B50] Mizuno R. Kawada K. Sakai Y. (2019). Prostaglandin E2/EP Signaling in the Tumor Microenvironment of Colorectal Cancer. Int. J. Mol. Sci. 20 (24), 6254. 10.3390/ijms20246254 PMC6940958 31835815

[B90] Mogensen T. H. (2009). Pathogen Recognition and Inflammatory Signaling in Innate Immune Defenses. Clin Microbiol Rev. 22 (2), 240–273. 10.1128/CMR.00046-08 19366914 PMC2668232

[B51] Nishihira J. Mitsuyama K. (2009). Overview of the Role of Macrophage Migration Inhibitory Factor (MIF) in Inflammatory Bowel Disease. Curr. Pharm. Des. 15 (18), 2104–2109. 10.2174/138161209788489113 19519441

[B52] Olén O. Erichsen R. Sachs M. C. Pedersen L. Halfvarson J. Askling J. (2020). Colorectal Cancer in Ulcerative Colitis: a Scandinavian Population-Based Cohort Study. Lancet 395 (10218), 123–131. 10.1016/S0140-6736(19)32545-0 31929014

[B53] Onizawa M. Nagaishi T. Kanai T. Nagano K. Oshima S. Nemoto Y. (2009). Signaling Pathway via TNF-alpha/NF-kappaB in Intestinal Epithelial Cells May Be Directly Involved in Colitis-Associated Carcinogenesis. Am. J. Physiol. Gastrointest. Liver Physiol. 296 (4), G850–G859. 10.1152/ajpgi.00071.2008 19179628

[B54] Pacheco-Fernández T. Juárez-Avelar I. Illescas O. Terrazas L. I. Hernández-Pando R. Pérez-Plasencia C. (2019). Macrophage Migration Inhibitory Factor Promotes the Interaction between the Tumor, Macrophages, and T Cells to Regulate the Progression of Chemically Induced Colitis-Associated Colorectal Cancer. Mediators Inflamm. 2019, 2056085. 10.1155/2019/2056085 31360118 PMC6652048

[B55] Parada Venegas D. De la Fuente M. K. Landskron G. González M. J. Quera R. Dijkstra G. (2019). Short Chain Fatty Acids (SCFAs)-Mediated Gut Epithelial and Immune Regulation and its Relevance for Inflammatory Bowel Diseases. Front. Immunol. 10, 277. 10.3389/fimmu.2019.00277 30915065 PMC6421268

[B56] Pastille E. Faßnacht T. Adamczyk A. Ngo Thi Phuong N. Buer J. Westendorf A. M. (2021). Inhibition of TLR4 Signaling Impedes Tumor Growth in Colitis-Associated Colon Cancer. Front. Immunol. 12, 669747. 10.3389/fimmu.2021.669747 34025672 PMC8138317

[B57] Peng C. Ouyang Y. Lu N. Li N. (2020). The NF-κB Signaling Pathway, the Microbiota, and Gastrointestinal Tumorigenesis: Recent Advances. Front. Immunol. 11, 1387. 10.3389/fimmu.2020.01387 32695120 PMC7338561

[B58] Popivanova B. K. Kitamura K. Wu Y. Kondo T. Kagaya T. Kaneko S. (2008). Blocking TNF-Alpha in Mice Reduces Colorectal Carcinogenesis Associated with Chronic Colitis. J. Clin. Invest. 118 (2), 560–570. 10.1172/JCI32453 18219394 PMC2213370

[B59] Qiu X. Ma J. Wang K. Zhang H. (2017). Chemopreventive Effects of 5-aminosalicylic Acid on Inflammatory Bowel Disease-Associated Colorectal Cancer and Dysplasia: a Systematic Review with Meta-Analysis. Oncotarget 8 (1), 1031–1045. 10.18632/oncotarget.13715 27906680 PMC5352032

[B60] Quandt J. Arnovitz S. Haghi L. Woehlk J. Mohsin A. Okoreeh M. (2021). Wnt-β-catenin Activation Epigenetically Reprograms Treg Cells in Inflammatory Bowel Disease and Dysplastic Progression. Nat. Immunol. 22 (4), 471–484. 10.1038/s41590-021-00889-2 33664518 PMC8262575

[B61] Rajamäki K. Taira A. Katainen R. Välimäki N. Kuosmanen A. Plaketti R.-M. (2021). Genetic and Epigenetic Characteristics of Inflammatory Bowel Disease-Associated Colorectal Cancer. Gastroenterology 161 (2), 592–607. 10.1053/j.gastro.2021.04.042 33930428

[B62] Richard M. L. Liguori G. Lamas B. Brandi G. da Costa G. Hoffmann T. W. (2018). Mucosa-associated Microbiota Dysbiosis in Colitis Associated Cancer. Gut Microbes 9 (2), 131–142. 10.1080/19490976.2017.1379637 28914591 PMC5989788

[B63] Risques R. A. Lai L. A. Himmetoglu C. Ebaee A. Li L. Feng Z. (2011). Ulcerative Colitis-Associated Colorectal Cancer Arises in a Field of Short Telomeres, Senescence, and Inflammation. Cancer Res. 71 (5), 1669–1679. 10.1158/0008-5472.CAN-10-1966 21363920 PMC3077943

[B64] Rutter M. Saunders B. Wilkinson K. Rumbles S. Schofield G. Kamm M. (2004). Severity of Inflammation Is a Risk Factor for Colorectal Neoplasia in Ulcerative Colitis. Gastroenterology 126 (2), 451–459. 10.1053/j.gastro.2003.11.010 14762782

[B65] Salesse L. Lucas C. Hoang M. H. T. Sauvanet P. Rezard A. Rosenstiel P. (2021). Colibactin-Producing *Escherichia coli* Induce the Formation of Invasive Carcinomas in a Chronic Inflammation-Associated Mouse Model. Cancers (Basel) 13 (9), 2060. 10.3390/cancers13092060 33923277 PMC8123153

[B66] Salk J. J. Salipante S. J. Risques R. A. Crispin D. A. Li L. Bronner M. P. (2009). Clonal Expansions in Ulcerative Colitis Identify Patients with Neoplasia. Proc. Natl. Acad. Sci. U S A. 106 (49), 20871–20876. 10.1073/pnas.0909428106 19926851 PMC2779829

[B67] Santaolalla R. Sussman D. A. Ruiz J. R. Davies J. M. Pastorini C. España C. L. (2013). TLR4 Activates the β-catenin Pathway to Cause Intestinal Neoplasia. PloS one 8 (5), e63298. 10.1371/journal.pone.0063298 23691015 PMC3653932

[B68] Santoru M. L. Piras C. Murgia A. Palmas V. Camboni T. Liggi S. (2017). Cross Sectional Evaluation of the Gut-Microbiome Metabolome axis in an Italian Cohort of IBD Patients. Sci. Rep. 7 (1), 9523. 10.1038/s41598-017-10034-5 28842640 PMC5573342

[B69] Scarpa M. Scarpa M. Castagliuolo I. Erroi F. Kotsafti A. Basato S. (2016). Aberrant Gene Methylation in Non-neoplastic Mucosa as a Predictive Marker of Ulcerative Colitis-Associated CRC. Oncotarget 7 (9), 10322–10331. 10.18632/oncotarget.7188 26862732 PMC4891122

[B70] Schmitt M. Greten F. R. (2021). The Inflammatory Pathogenesis of Colorectal Cancer. Nat. Rev. Immunol. 21 (10), 653–667. 10.1038/s41577-021-00534-x 33911231

[B71] Sheng Y. H. Giri R. Davies J. Schreiber V. Alabbas S. Movva R. (2021). A Nucleotide Analog Prevents Colitis-Associated Cancer via Beta-Catenin Independently of Inflammation and Autophagy. Cell Mol Gastroenterol Hepatol 11 (1), 33–53. 10.1016/j.jcmgh.2020.05.012 32497793 PMC7593585

[B72] Soetikno R. M. Lin O. S. Heidenreich P. A. Young H. S. Blackstone M. O. (2002). Increased Risk of Colorectal Neoplasia in Patients with Primary Sclerosing Cholangitis and Ulcerative Colitis: a Meta-Analysis. Gastrointest. Endosc. 56 (1), 48–54. 10.1067/mge.2002.125367 12085034

[B73] Somineni H. K. Venkateswaran S. Kilaru V. Marigorta U. M. Mo A. Okou D. T. (2019). Blood-Derived DNA Methylation Signatures of Crohn's Disease and Severity of Intestinal Inflammation. Gastroenterology 156 (8), 2254. 10.1053/j.gastro.2019.01.270 30779925 PMC6529254

[B74] Sorbara M. T. Dubin K. Littmann E. R. Moody T. U. Fontana E. Seok R. (2019). Inhibiting Antibiotic-Resistant Enterobacteriaceae by Microbiota-Mediated Intracellular Acidification. J. Exp. Med. 216 (1), 84–98. 10.1084/jem.20181639 30563917 PMC6314524

[B75] Suarez-Lopez L. Kong Y. W. Sriram G. Patterson J. C. Rosenberg S. Morandell S. (2020). MAPKAP Kinase-2 Drives Expression of Angiogenic Factors by Tumor-Associated Macrophages in a Model of Inflammation-Induced Colon Cancer. Front. Immunol. 11, 607891. 10.3389/fimmu.2020.607891 33708191 PMC7940202

[B76] Swidsinski A. Ladhoff A. Pernthaler A. Swidsinski S. Loening-Baucke V. Ortner M. (2002). Mucosal flora in Inflammatory Bowel Disease. Gastroenterology 122 (1), 44–54. 10.1053/gast.2002.30294 11781279

[B77] Tan J. McKenzie C. Potamitis M. Thorburn A. N. Mackay C. R. Macia L. (2014). The Role of Short-Chain Fatty Acids in Health and Disease. Adv. Immunol. 121, 91–119. 10.1016/B978-0-12-800100-4.00003-9 24388214

[B78] Terdiman J. P. Steinbuch M. Blumentals W. A. Ullman T. A. Rubin D. T. (2007). 5-Aminosalicylic Acid Therapy and the Risk of Colorectal Cancer Among Patients with Inflammatory Bowel Disease. Inflamm. Bowel Dis. 13 (4), 367–371. 10.1002/ibd.20074 17206695

[B79] Tian Y. Xu Q. Sun L. Ye Y. Ji G. (2018). Short-chain Fatty Acids Administration Is Protective in Colitis-Associated Colorectal Cancer Development. J. Nutr. Biochem. 57, 103–109. 10.1016/j.jnutbio.2018.03.007 29694938

[B80] Ullman T. A. Itzkowitz S. H. (2011). Intestinal Inflammation and Cancer. Gastroenterology 140 (6), 1807–1816. 10.1053/j.gastro.2011.01.057 21530747

[B81] Wang C. Gong G. Sheh A. Muthupalani S. Bryant E. M. Puglisi D. A. (2017). Interleukin-22 Drives Nitric Oxide-dependent DNA Damage and Dysplasia in a Murine Model of Colitis-Associated Cancer. Mucosal Immunol. 10 (6), 1504–1517. 10.1038/mi.2017.9 28198364 PMC5557711

[B82] Wang W. Jovel J. Halloran B. Wine E. Patterson J. Ford G. (2015). Metagenomic Analysis of Microbiome in colon Tissue from Subjects with Inflammatory Bowel Diseases Reveals Interplay of Viruses and Bacteria. Inflamm. Bowel Dis. 21 (6), 1419–1427. 10.1097/MIB.0000000000000344 25939040 PMC4450971

[B83] West N. R. Hegazy A. N. Owens B. M. J. Bullers S. J. Linggi B. Buonocore S. (2017). Oncostatin M Drives Intestinal Inflammation and Predicts Response to Tumor Necrosis Factor-Neutralizing Therapy in Patients with Inflammatory Bowel Disease. Nat. Med. 23 (5), 579–589. 10.1038/nm.4307 28368383 PMC5420447

[B84] Wijnands A. M. de Jong M. E. Lutgens M. W. M. D. Hoentjen F. Elias S. G. Oldenburg B. (2021). Prognostic Factors for Advanced Colorectal Neoplasia in Inflammatory Bowel Disease: Systematic Review and Meta-Analysis. Gastroenterology 160 (5), 1584–1598. 10.1053/j.gastro.2020.12.036 33385426

[B85] Wilson J. A. (2008). Tumor Necrosis Factor Alpha and Colitis-Associated colon Cancer. N. Engl. J. Med. 358 (25), 2733–2734. 10.1056/NEJMcibr0803116 18565868

[B86] Yaeger R. Shah M. A. Miller V. A. Kelsen J. R. Wang K. Heins Z. J. (2016). Genomic Alterations Observed in Colitis-Associated Cancers Are Distinct from Those Found in Sporadic Colorectal Cancers and Vary by Type of Inflammatory Bowel Disease. Gastroenterology 151 (2), 278–e6. 10.1053/j.gastro.2016.04.001 27063727 PMC5472377

[B87] Yang Y. Gharaibeh R. Z. Newsome R. C. Jobin C. (2020). Amending Microbiota by Targeting Intestinal Inflammation with TNF Blockade Attenuates Development of Colorectal Cancer. Nat. Cancer 1 (7), 723–734. 10.1038/s43018-020-0078-7 33768208 PMC7990316

[B88] Zhang Y. Weng Y. Gan H. Zhao X. Zhi F. (2018). Streptococcus Gallolyticus Conspires Myeloid Cells to Promote Tumorigenesis of Inflammatory Bowel Disease. Biochem. Biophys. Res. Commun. 506 (4), 907–911. 10.1016/j.bbrc.2018.10.136 30392911

[B89] Zhu Z. Mei Z. Guo Y. Wang G. Wu T. Cui X. (2018). Reduced Risk of Inflammatory Bowel Disease-Associated Colorectal Neoplasia with Use of Thiopurines: a Systematic Review and Meta-Analysis. J. Crohns Colitis 12 (5), 546–558. 10.1093/ecco-jcc/jjy006 29370346

